# Usefulness of paired samples for the Serodiagnosis of toxoplasmosis infection in a tertiary teaching Hospital in Malaysia

**DOI:** 10.1186/s12879-019-3830-9

**Published:** 2019-02-28

**Authors:** Padmaloseni Thangarajah, Khalid Hajissa, Weng Kin Wong, Muhammad Amiruddin Abdullah, Nabilah Ismail, Zeehaida Mohamed

**Affiliations:** 1 Department of Microbiology, Melaka Hospital, 75400 Melaka, Malaysia; 2 Department of Medical Microbiology & Parasitology, School of Medical Sciences, Universiti Sains Malaysia, 16150 Kubang Kerian, Kelantan Malaysia; 3 School of Health Sciences, Universiti Sains Malaysia, 16150 Kubang Kerian, Malaysia; 4 Hospital Universiti Sains Malaysia, 16150 Kubang Kerian, Kelantan Malaysia

**Keywords:** Toxoplasmosis, Paired samples, Serodiagnosis, Malaysia

## Abstract

**Background:**

Accurate diagnosis of *Toxoplasma gondii* (*T. gondii)* infection remains elusive and requires a comprehensive assessment through laboratory and clinical investigation. In this study, a diagnostic algorithm based on paired serum samples and clinical data was developed and evaluated.

**Methods:**

A total of 1267 suspected cases of *Toxoplasma* infection were enrolled in this study from January 2016 to December 2016. The cases were screened for anti-*Toxoplasma* IgM and IgG by electrochemiluminiscence immunoassay (ECLIA) method. Based on the serological profiles, all cases with first seropositive serum samples were considered as suggestive cases of *Toxoplasma* infection. Thus, second serum samples were obtained after an interval of 2 weeks. The diagnosis was made based on laboratory results and clinical data.

**Results:**

A total of 482 *T. gondii* seroreactive cases were selected. The patient’s records were traced and the data were analysed. Accordingly, 152 cases were diagnosed as clinically confirmed cases; 198 cases were clinically asymptomatic and 132 cases were newborn babies or infants who did not have toxoplasmosis and only acquired passive immunity from their mothers. The paired serum algorithm allowed classifying the seroreactive cases as follows: early (0.6%), acute (1.9%), reactivation (13.5%), recent (1.5%), passive immunity from mother (27.3%) and possible congenital infections (1.2%). In addition, cases of reactivated toxoplasmosis were detected among the pregnant mothers (13/82; 15.8%), children aged above 1 year (2/8; 25.0%) and immunocompetent mothers (5/135; 3.7%). Furthermore, the application of the paired serum analysis resulted in remarkably improved treatment initiation.

**Conclusions:**

Toxoplasmosis diagnosis and treatment can be improved through the use of paired serum diagnostic algorithm.

## Background

Toxoplasmosis is a common parasitic disease caused by *Toxoplasma gondii*, an obligate unicellular coccidian parasite [1]. The parasite infects more than 30% of the global population, even though, most infected individuals do not develop any clinical symptoms, and the disease often remains unrecognised [2]. However, severe clinical complications, such as toxoplasmic encephalitis, might occur in immunocompromised individuals, including HIV patients and organ transplant recipients. Moreover, an infection during pregnancy can cause congenital defects, spontaneous abortion and severe foetal abnormalities [3, 4].

The accurate diagnosis of the disease is crucial because early instituted treatment remarkably improves clinical outcomes and decreases mother-to-child transmission in congenital infections [5]. Serological assays are the primary approach for achieving satisfactory results and play a crucial role in disease management [1]. The results of serological tests, especially ELISA, are generally well accepted by clinicians owing to their high sensitivities and specificities, relatively low costs and fast results [6]. Regardless of the clinical sign, detecting toxoplasma antibodies is the mainstay of toxoplasmosis diagnosis and is generally used for routine investigation in many medical centres worldwide [7]. The fundamental process for identifying *T. gondii* infection is usually based on the detection of serum IgMs or IgGs or both. However, the presence of IgMs only suggests current or active infection, whereas the presence of IgGs only indicates chronic or past infection [8].

Hence, understanding the kinetics of antibody response is crucial in the development of a diagnostic strategy. *T. gondii*-specific IgMs are the first secreted antibodies that can be detected from 5 days to several weeks after infection. The levels of these antibodies rise, peak at the second month and decline from 6 to 9 months after infection [2]. However, these antibodies can be detected for as long as 2 years or more after acute infection without any clinical significance, thus complicating the interpretation of serological results [9, 10]. Moreover, the presence of *T. gondii*-specific IgM is not always indicative of a recent infection. Meanwhile, *T. gondii*-specific IgGs appear within 1–2 weeks after infection; the levels of these antibodies peak from 12 weeks to 6 months and persist for decades and even for a lifetime [11]. Positive IgG results are not indicative of infection status (recent and latent). Recent studies have emphasised that single serum sample analysis is suboptimal for the precise diagnosis of toxoplasmosis. The confirmation or exclusion of *T. gondii* infection can be achieved precisely only with correlated clinical and laboratory information [2, 12]. Current diagnostic algorithms must be improved for the accurate distinction among early, acute, reactivation, recent, latent and possible congenital infections. Therefore, the present study aims to evaluate the application of a paired serum diagnostic algorithm and clinical data and to establish such algorithm as a new diagnostic model for the laboratory investigation of *Toxoplasma* infection in a tertiary teaching hospital. Serological results were interpreted on the basis of the diagnostic flowcharts of the model.

## Methods

### Study participants

A total of 1267 hospitalised patients with clinically suspected cases of *Toxoplasma* infection admitted to Hospital Universiti Sains Malaysia (HUSM) from January 2016 to December 2016 were enrolled in this study. HUSM is a tertiary teaching hospital at the northeast Peninsular Malaysia.

### Sera collection and laboratory investigation

Sera collected from the patients were serologically screened for anti-*Toxoplasma* IgMs and IgGs with an Elecsys toxo IgG and IgM immunoassays (Roche, Germany) in accordance with manufacturer’s instructions. The IgG results were expressed in international unit (IU). Based on the kit interpretation, sera with IgG titres of < 1 IU/mL were considered non-reactive for anti-*T. gondii* IgGs, whereas sera with IgG titres of 1–30 IU/mL and > 30 IU/ml were considered indeterminate and reactive, respectively. Toxo IgM results were expressed through the cut-off index (COI – sample signal/cut off). Samples with COI of < 0.8 were classified as non-reactive. Samples with COI of ≥0.8 but < 1.0 were classified as indeterminate, whereas samples with COI of ≥1.0 were considered IgM reactive.

### Diagnostic algorithm based on paired serum sample

**Fig. 1:**
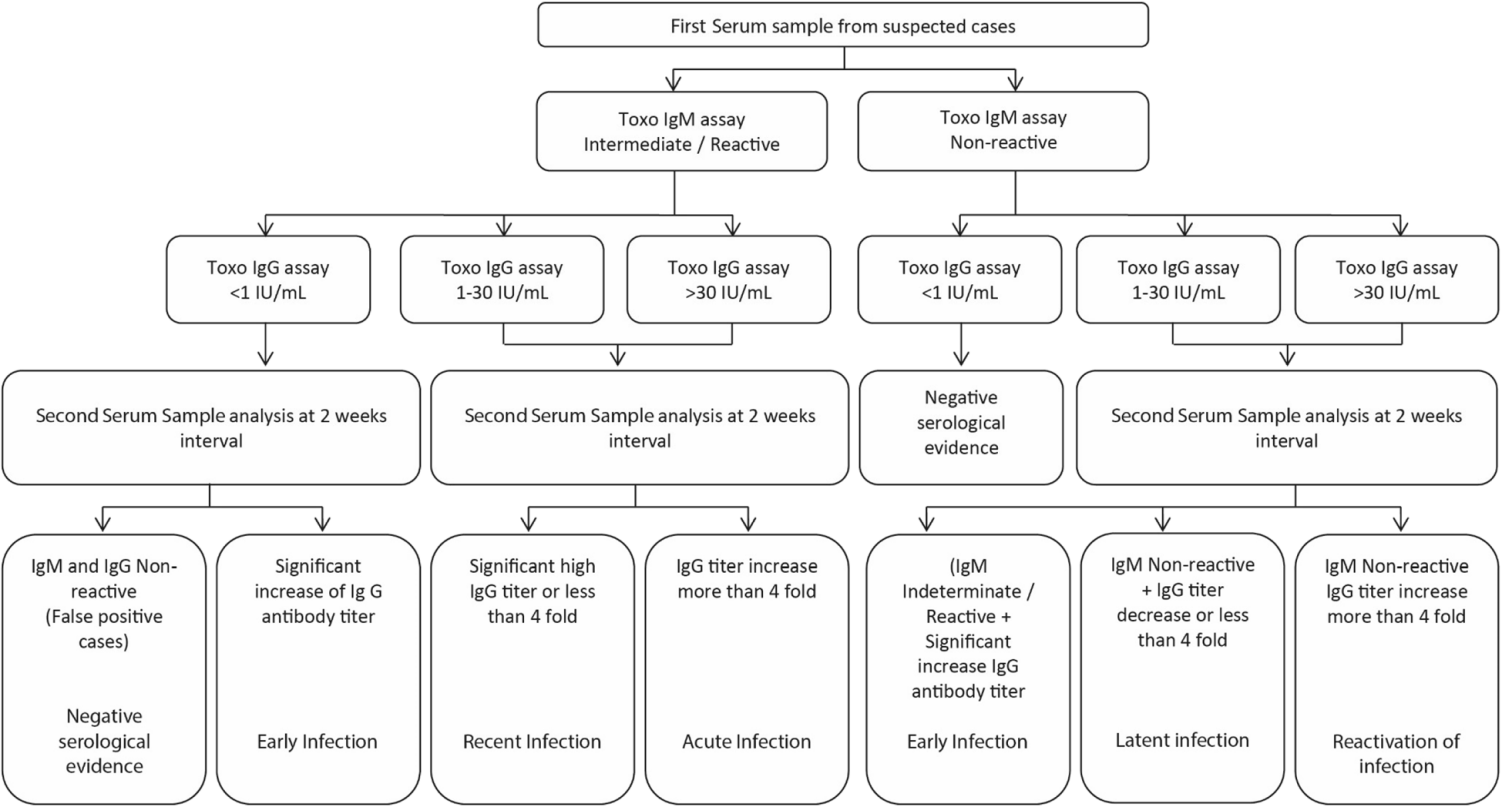
Algorithm for the serodiagnosis of toxoplasmosis. < 1 IU/ml = Non-reactive, 1 –30 IU/ml = Indeterminate, > 30 IU/ml = Reactive

All the sera were preliminary screened for anti-*Toxoplasma* IgMs and IgGs. Second serum samples were collected at 2 weeks interval from cases with first serum samples that were suggestive of *Toxoplasma* infection. For diagnosis and infection classification, the serological profiles of 482 cases were analysed and matched with the clinical data that were obtained from the records of patients. While, a total of 785 cases were excluded. The exclusion criteria were as follows: patients with *Toxoplasma* IgM and IgG non-reactive sera, patients with single serum samples, patients who passed away after the collection of the first serum samples, newborn babies or infants with IgG reactive sera but their mothers’ serum samples were not available, patients already initiated treatment prior to first serum sample collection, second serum sample was sent at more than 3 weeks duration or repeated serum samples were sent during the 2 weeks interval and patients with missed clinical folders. The diagnostic algorithm applied in this study is shown in Fig. 1.

### Serological interpretation and classification of infection

Basing on the critical review and kinetics of antibody response, the serological interpretation was generated and the results were used to classify the *Toxoplasma* infections as early, acute, reactivation, recent, latent, possible congenital infection or possible cross immunity from mother (Fig. 1). In addition, among newborn babies and infant, mother’s serum sample were requested as paired serum sample to correlate the evidence of IgG antibody as passive immunity from mother.

### Statistical analysis

Data were recorded and analysed with SPSS version 24.0. Categorical data were presented in frequency and percentage. The distribution of the categorical data between the two groups was analysed with Pearson’s chi-square test.

## Results

**Fig. 2:**
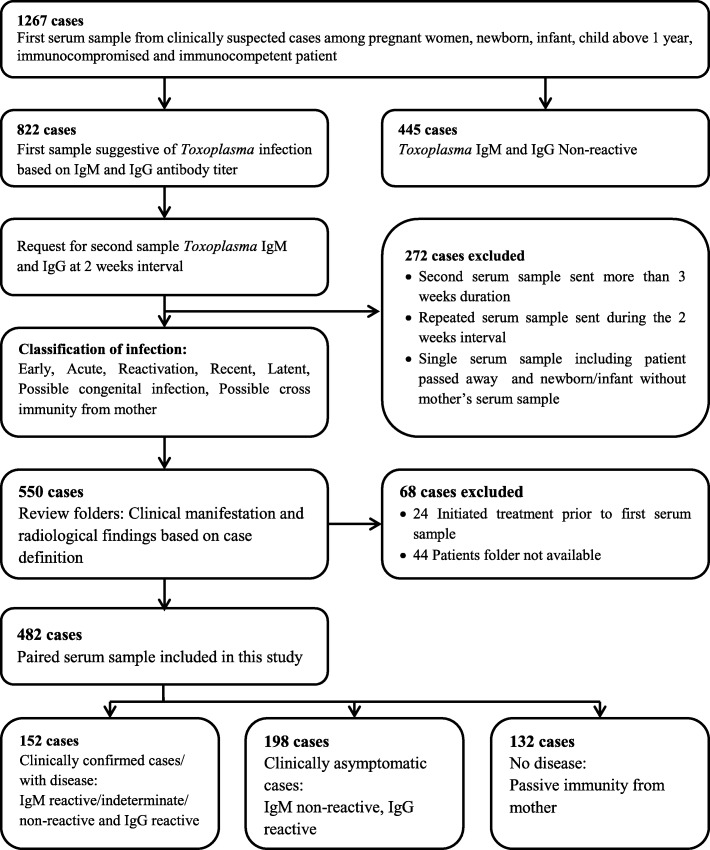
Results of the case selection process from the conceptual framework

The screening results of the first serum samples for *Toxoplasma* IgMs and IgGs indicated that the overall seroprevalence of toxoplasmosis antibodies in the suspected cases was 64.9% (822/1267). Second serum samples from 822 patients were requested at an interval of 2 weeks. Only 482 cases (82 cases: pregnant women; 141 cases: new born babies and infants; 8 cases:children aged above 1 year; 66 cases: immunocompromised; 50 cases: immunocompetent with ocular toxoplasmosis and 135 cases: immuneocompetent mothers) fulfilled the inclusion criteria and were subjected to further analysis, and the records of these patients were traced. By comparing the serological profiles of the first serum samples with the corresponding second serum samples the infection was classified as early, acute, reactivation, recent, latent, possible congenital infection or possible cross-immunity from mother. The clinical data of the patients, particularly signs and symptoms and radiological or ophthalmological findings, were obtained from the clinicians’ notes and matched with the serological results for the categorisation of the cases into three groups: clinically confirmed with disease (*n* = 152), clinically asymptomatic (*n* = 198) and newborn babies or infants with passive immunity from mother (*n* = 132) (Fig. 2).

Based on the single serum sample analysis results which are currently applied in many clinics the pattern of *Toxoplasma* infection in the study participants; only 3.3% were acute cases and 96.7% were chronic cases. After the incorporation of second serum analysis results and clinical data, 18.7% were suspected to be active cases, that is, the cases were early, acute, reactivation, recent and possible congenital infections (Table 1). Based on the single serum sample algorithm, 10 cases of acute infection and 47 cases of chronic infection require treatment. However, the paired serum sample algorithm revealed a higher number (56/482, 11.6%) of patients who received treatment and who showed serological evidence that was suggestive of active infection than the single serum sample algorithm (10/482, 2.1%).

**Table 1:** Clinical classification of cases based on to single and paired serum sample algorithm

Study Group	n	Single Serum Sample, n (%)		Paired Serum Sample, n (%)						
		Acute	Chronic	Early	Acute	Reactivation	Recent	Possible congenital infection	Passive immunity from mother	Latent
Clinical Classification based on Serological Findings										
Pregnant Women	82	7 (8.5)	75 (91.5)	–	2 (2.4)	13 (15.8)	5 (6.1)	–	–	62 (75.6)
Newborn and Infant	141	3 (2.1)	138 (97.9)	–	3 (2.1)	–	–	6 (4.3)	132 (93.6)	–
Child > 1 years old	8	–	8 (100)	–	–	2 (25.0)	–	–	–	6 (75.0)
Immunocompromised	66	2 (3.0)	64 (97.0)	–	2 (3.0)	29 (43.9)	–	–	–	35 (53.0)
Ocular Toxoplasmosis	50	2 (4.0)	48 (96.0)	2 (4.0)	1 (2.0)	16 (32.0)	1 (2.0)	–	–	30 (60.0)
Mother	135	2 (1.5)	133 (98.5)	1 (0.7)	1 (0.7)	5 (3.7)	1 (0.7)	–	–	127 (94.1)
Suggestive of active cases	482	Yes	No	Yes	Yes	Yes	Yes	Yes	No	No
		16 (3.3)	466 (96.7)	90 (18.7)					392 (81.3)	
Treatment Initiation based on Serological and Clinical Findings										
Pregnant Women	82	2/7	1/75	–	2/2	2/13	0/5	–	–	0/62
Newborn and Infant	141	3/3	0/138	–	3/3	–	–	0/6	0/132	–
Child > 1 years old	8	–	1/8	–	–	1/2	–	–	–	0/6
Immunocompromised	66	2/2	29/64	–	2/2	29/29	–	–	–	2/35
Immunocompetent	185	3/4	16/181	3/3	2/2	11/21	1/2	–	–	3/157
Ocular	50	2/2	16/48	2/2	1/1	11/16	1/1	–	–	3/30
Mother	135	1/2	0/133	1/1	1/1	0/5	0/1	–	–	0/127
Treatment initiation based on serology and clinical findings	482	10 (2.1)	47 (9.8)	56 (11.6)					5 (1.0)	

## Discussion

Toxoplasmosis is a silent threat to pregnant women, newborn babies, infants, children, immunocompromised patients and immunocompetent individuals. Serological tests are the primary approach for achieving satisfactory results and play a crucial role in the screening and conduct of follow-up [3, 13]. The fundamental process of toxoplasmosis serology relies on the detection of anti-*Toxoplasma gondii*-specific IgMs and IgGs. However, differentiating between acute (recently acquired infection) and chronic infections remains a challenge to clinicians and clinical diagnostic centres. The observed persistence of IgMs from 1 week to several months or years after infection complicates the interpretation of acute infection. The detected IgG antibodies years after infection do not provide any information about the time of infection [14]. Initially, the spectrum of *Toxoplasma* infection was divided into acute and chronic infection, but this mode of classification cannot be used for the identification of reactivated infection [15]. Moreover, serological results based on IgM and IgG profiles are often difficult to interpret and provide insufficient evidence of an acute or chronic disease, especially when only a single serum sample is tested. Hence, using the serological data of patients and clinical information can be an effective strategy for improving disease diagnosis. The French National Reference Centre for Toxoplasmosis recommended a practical approach based on the routine and clinical situation [2]. In this study, a paired serum diagnostic algorithm was established as a diagnostic model for the laboratory investigation of *Toxoplasma* infection and clinical information was used. This model is expected to provide an effective scheme for classifying infection.

Using the algorithm, we found that 38% (482/1267) of the patients were seroreactive for *Toxoplasma* infection. A slightly higher seroprevalence of toxoplasmosis (44.2%) was reported previously from the same hospital [16]. However, the previous study did not distinguish acute infections from chronic infections. By contrast, the proposed paired serum diagnostic algorithm allows further disease classification, which is an important factor for treatment initiation. In some cases, treatment was started according to the clinical indication supported by the first *Toxoplasma* IgM and high IgG titre. The treatment was not based on infection classification because the search for substantial rise or seroconversion in IgG titre requires 2 weeks and thus may delay the decision-making process of clinician and might worsen a patient’s condition. Meanwhile, the number of patients who received treatment increased (11.6%) when the paired serum algorithm was used and was higher than value obtained with the single serum algorithm. This result suggests that only about 2.1% of the patients have serological evidence that is suggestive of active infection. Moreover, pairing the serum of the mother with the serum of a newborn baby or infant in suspected cases of congenital toxoplasmosis led to the true diagnosis. Hence, misleading treatment initiation and unwanted follow-up for the newborn baby or infant can be prevented.

Several attempts to distinguish acute from chronic infections were reported, but no conclusive results have been obtained [17, 18]. Initially, the spectrum of *Toxoplasma* infection was divided into acute and chronic infection; unfortunately, this mode of classification cannot be used in the identification of reactivated infection [17]. By focusing on antibody titre and understanding the kinetics of antibody responses, this study provided additional schemes for infection classification. The interpreted serological results were used in the classification of infection. Thus, in pregnant women, children who were > 1 years and immunocompetent mothers, 13 (15.8%), 2 (25.0%) and 5 (3.7%) were identified as cases of reactivated infection, respectively.

This study suggested a preliminary model for classifying *Toxoplasma* infection. However, accurate differentiation between infection stages remains a challenge. A tool that allows such differentiation is not yet available [1, 17]. The identification of stage-specific antigens might be rewarding because the overlapping of immunological responses in two parasitic stages is likely to occur [17]. Additionally, IgG avidity can be used for detecting acute or chronic infection because promising results were obtained in various studies [2, 19, 20]. However, IgG avidity is not recommended for the diagnosis of reactivation cases because it cannot be used for measuring antibody titre. By contrast, the paired serum algorithm can be used for identifying cases of reactivated infection.

## Conclusion

In conclusion, this study shows that the toxoplasmosis diagnosis can be improved by using the paired serum analysis mode. A clear classification of toxoplasmosis status is useful to physicians and treatment initiation especially for high risk and vulnerable groups.

## Abbreviations

COI: cut-off index
ECLIA: Electrochemiluminiscence immunoassay
ELISA: Enzyme-linked Immunosorbent Assay
HUSM: Hospital Universiti Sains Malaysia
IU: International unit
